# High insulinemic potential of diet and lifestyle is associated with increased risk of chronic kidney disease incident in adults

**DOI:** 10.1186/s12882-023-03059-8

**Published:** 2023-01-09

**Authors:** Hossein Farhadnejad, Farshad Teymoori, Mitra Kazemi Jahromi, Golaleh Asghari, Ebrahim Mokhtari, Parvin Mirmiran, Fereidoun Azizi

**Affiliations:** 1 Nutrition and Endocrine Research Center, Research Institute for Endocrine Sciences, Shahid Beheshti University of Medical Sciences, Tehran, Iran; 2 Department of Nutrition, School of Public Health, Iran University of Medical Sciences, Tehran, Iran; 3 Endocrinology and Metabolism Research Center, Hormozgan University of Medical Sciences, Bandar Abbas, Iran; 4 Department of Clinical Nutrition and Dietetics, Faculty of Nutrition Sciences and Food Technology, National Nutrition and Food Technology Research Institute, Shahid Beheshti University of Medical Sciences, Tehran, Iran; 5 Endocrine Research Center, Research Institute for Endocrine Sciences, Shahid Beheshti University of Medical Sciences, Tehran, Iran

**Keywords:** Dietary pattern, Lifestyle, Insulinemic indices, Chronic kidney disease

## Abstract

**Background:**

The role of higher insulinemic effects of dietary pattern and lifestyle factors on the risk of chronic kidney disease (CKD) is not well-studied. In the current study, we aimed to investigate the relationship between the insulinemic potential of diet and lifestyle with the risk of CKD in adults.

**Methods:**

A total of 6044 individuals without CKD, aged>18 years, were recruited from among participants of the Tehran Lipid and Glucose Study (third and fourth surveys) and followed a mean of 6.03 years(follow-up rate:94.95%). The dietary intake data were collected using a food frequency questionnaire. The insulinemic potential of diet and lifestyle was determined based on four empirical indices, including the empirical dietary index for hyperinsulinemia (EDIH), the empirical dietary index for insulin resistance (EDIR), the empirical lifestyle index for hyperinsulinemia (ELIH), and the empirical lifestyle index for insulin resistance (ELIR).

**Results:**

Mean ± SD age of all study participants (54.3% women) was 37.8 ± 12.8 years. During the 6.03 years of follow-up (46,889.8 person-years), 1216(20.1%) new cases of CKD were identified. According to the multivariable-adjusted model, the risk of CKD incident is increased across quintiles of EDIR (OR = 1.29;95% CI: 1.06–1.57), ELIH (OR = 1.35; 95%CI: 1.10–1.67), and ELIR (OR = 1.24; 95%CI:1.02–1.51). However, no significant relationship was found between the EDIH score and the risk of CKD.

**Conclusion:**

Results of the current study showed that dietary pattern with a high EDIR score and a lifestyle with higher ELIH and ELIR scores may be related to increasing the risk of CKD incident. However, no significant association was observed between EDIH score and CKD incident.

## Background

Chronic kidney disease (CKD) is one of the major contributors to the global burden of disease via increasing cardiovascular disease risk and mortality worldwide [1]. CKD is characterized by a substantial and progressive change in glomerular filtration rate (GFR) caused by structural and functional damage lasting for more than 3 months. The latest report on the Global Burden of Disease in 2017 indicated that CKD accounts for 1.2 million deaths worldwide; in addition, 7.6% of deaths due to cardiovascular diseases could be attributed to kidney dysfunction [1, 2]. Various risk factors, including weight gain, hypertension (HTN), type 2 diabetes mellitus (T2DM), and an unhealthy lifestyle, positively influence the occurrence of CKD [3, 4]. Correspondingly, evidence suggests the protective role of lifestyle modifications such as body fat reduction, increased physical activity, and nutritional manipulations on preventing or reducing CKD progression [5, 6].

Recently, it has been reported that hyperinsulinemia and insulin resistance (IR), two insulin homeostasis-related disorders, play a destructive role in the pathogenesis of kidney disease and other chronic metabolic diseases. A review of animal studies has shown that hyperinsulinemia and IR may cause kidney damage by increasing albumin excretion, glomerular hyperfiltration, endothelial dysfunction, and incrementing the risk of kidney fibrosis [7, 8]. Given the importance of hyperinsulinemia and IR as predisposing factors in the incidence of metabolic diseases, several studies have evaluated the role of nutrition and other lifestyle factors, such as physical activity and obesity, in the pathogenesis of these insulin homeostasis-related disorders and metabolic disorders, with different aspects [9–14]. In this regard, some studies have determined the insulinemic potential of dietary pattern and lifestyle and investigated its effects on increasing the risk of IR and hyperinsulinemia and subsequent chronic diseases.

Tabung et al. have recently introduced the insulinemic potential of diet and lifestyle [15], which has been determined based on four insulinemic indices, including the empirical dietary indexes for hyperinsulinemia (EDIH), the empirical dietary indexes for IR (EDIR), the empirical lifestyle indices for hyperinsulinemia (ELIH), and empirical lifestyle indices for IR (ELIR). To date, no study has examined the association of the insulinemic potential of diet and lifestyle, including EDIH, ELIH, EDIR, and ELIR, with the risk of CKD development, some studies have suggested that adherence to lifestyle and dietary pattern with a higher score of the above-mentioned insulinemic indices may be associated with an increased risk of some metabolic diseases as predisposing factors for CKD risk, such as T2D and obesity, and also various types of cancer [16–22].

Given the possible adverse effect of hyperinsulinemia and IR on the pathogenesis of kidney disease and the lack of data on the role of the above-mentioned insulinemic indices in the development of CKD risk, in the present study, we aimed to investigate the relationship between the insulinemic potential of lifestyle and dietary pattern and the risk of CKD in the adult population.

## Materials and methods

### Study participants

#### Tehran lipid and glucose study

The current study was performed in the framework of the Tehran Lipid and Glucose Study (TLGS), a population-based cohort study conducted to investigate the risk factors of chronic diseases among a representative urban population of Tehran, including 15,005 participants aged ≥ 3 years [23]. The first survey of TLGS was initiated in March 1999, and data collection conducted prospectively at 3 years intervals is ongoing. The baseline survey was a cross-sectional study conducted from 1999 to 2001, and surveys II (2002–2005), III (2006–2008), IV (2009–2011), V (2012–2015), and VI (2015–2018) were prospective follow-up surveys. The details of the TLGS have been explained previously [23]. In the third survey of the TLGS (2006–08), 3568 subjects were randomly selected for dietary assessment. Also, in the fourth survey of the TLGS (2009–2011), 7956 participants randomly selected subjects agreed to complete the dietary assessment.

#### Cohort entry

**Fig. 1:**
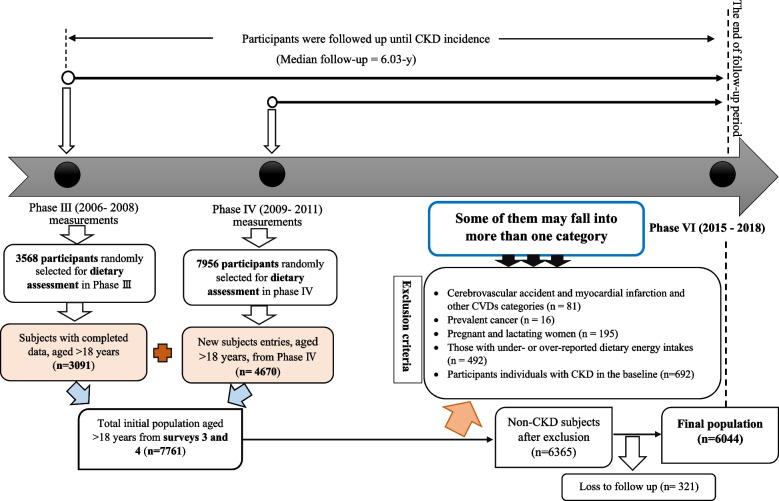
Flowchart of study population

For the current study, adult participants (aged > 18 years) of the third examination of TLGS with complete nutritional data (*n* = 3091) and also the new entries adult participants with complete nutritional data in the fourth examination (*n* = 4670) were enrolled (total initial population = 7761). Participants with a cardiovascular accident and myocardial infraction (*n* = 81), prevalent cancer (*n* = 16), pregnant and lactating women (*n* = 195), those with under or over-reported dietary energy intakes (out of the range 800–4200 kcal/d) (*n* = 492), and participants with CKD in the baseline (*n* = 692) were excluded. Some of them may fell into more than one category. Of 6365 CKD-free participants at baseline, who were followed up to the fourth (individuals who entered the study in phase 3 as the starting point of the study), fifth, and sixth examination of TLGS, 321 were lost to follow-up, and 6044 remained for final analysis (follow-up rate: 94.95%), (Fig. 1).

Evaluation of the CKD incidence status after the baseline assessment was conducted in the fourth, fifth, and sixth examinations using the one serum creatinine measurement and GFR calculation (for those who entered the study during the fourth phase as the base phase, CKD incidence status was assessed in the fifth or sixth phases). The first diagnosis of CKD was recorded as CKD incidence, and the follow-up times were calculated based on the first time of CKD diagnosis. For participants that remained healthy, their last CKD assessment was considered for the calculation of the follow-up period.

#### Physical activity assessment

The participant’s physical activity information was collected using a modifiable activity questionnaire (MAQ), previously modified and validated among Iranian adults [24]. Participants were asked to report and identify the frequency and time spent on activities of light, moderate, hard, and very hard intensity, over the past year, based on a list of common activities of daily life; total physical activity was presented as metabolic equivalent/hours per week (Met.h.wk).

#### Demographic, anthropometric, and lifestyle measures

Trained professional interviewers used a standard questionnaire to determine study population data on socio-demographic characteristics of participants (age [years], sex, education level [high school and diploma, academic education]), smoking habit, medical history, and medications through face-to-face interviews at baseline. The smoking habit in subjects was defined according to World Health Organization guidelines [25]. In the TLGS questionnaire, smoking was classified into yes/no groups; ‘yes’ defined subjects who smoked daily or occasionally or ex-smokers, and ‘no’ described the individuals who were non-smoked.

We used a standardized mercury sphygmomanometer with an accuracy of 2 mmHg to measure the systolic blood pressure (SBP) and diastolic blood pressure (DBP). All blood pressure measurement was performed for each participant twice on the right arm with a minimum interval of 30 s after a 15-min rest sitting on a chair; we considered the mean of the two measurements to be the participants’ blood pressure.

We measured participants’ body weight using a digital scale (Seca 881, Germany) to the nearest 100 g while the participants were in light clothes and without shoes. Height was measured by a stadiometer in a standing position without shoes and recorded to the nearest 0.5 cm. body mass index (BMI) was computed as weight (kg) divided by the height squared (m2). We also measured the waist circumference (WC) with an unstretched shape tape meter and recorded it to the nearest 0.1 cm. WC measurements were fulfilled at the abdominal and umbilical levels, over light clothing, and without any pressure on the body surface.

#### Biochemical measurements

The biochemical variables, including fasting blood glucose (FPG), 2-h blood glucose, and serum creatinine, were measured in participants. Based on the standard protocol, participants’ blood samples were taken after 12–14 h of overnight fasting in a sitting position and centrifuged within 30–45 min of collection. We performed all blood analyses at the TLGS research laboratory and used the Selectra 2 auto-analyzer (Vital Scientific, Spankeren, The Netherlands) to analyze the samples. FPG was determined using an enzymatic colorimetric method with glucose oxidase. Both inter-and intra-assay coefficient variations were 2.2% for FPG. For the oral glucose tolerance test, 82.5 g of glucose monohydrate solution (equivalent to 75 g anhydrous glucose) was administered orally to participants aged > 20 years. A second blood sample was taken 2-h after glucose ingestion. Serum creatinine concentration was measured using the standard colorimetric Jaffe Kinetic reaction method. Both intra-and inter-assay coefficients of variations (CVs) were < 3.1%. We performed all analyses using commercial kits (Pars Azmoon Inc., Tehran, Iran).

#### Definitions

##### Hypertension

Hypertension was determined in participants based on SBP/DBP ≥140/90 mmHg for individuals aged < 60 years and SBP/DBP ≥150/90 mmHg for those aged ≥60 years or using current antihypertensive medication [26].

##### Type 2 diabetes

The criteria of the American Diabetes Association (ADA) was used to define type 2 diabetes in participants according to the following criteria: FPG ≥ 126 mg/dl or 2-h post 75-g glucose load≥200 mg/dl or current anti-diabetes drug uses [27].

##### Chronic kidney disease

For the definition of CKD, the Epidemiology Collaboration (EPI) equation formula [28] was used to calculate eGFR based on one creatinine measurement in participants. We expressed the eGFR in ml/min/1.73m^2^ of body surface area. CKD was defined based on participants’ eGFR levels using the national kidney foundation guidelines as follows: eGFR≥60 ml/min/1.73m^2^ as not having CKD and eGFR< 60 ml/min/1.73m^2^ as having CKD.

#### Dietary intake assessment

The participant’s data on dietary intakes in the preceding year were collected using a valid and reliable 168- item semi-quantitative food frequency questionnaire (FFQ) with standard serving sizes [29, 30]. A trained nutritionist with at least 5 years’ experience in TLGS asked participants to report their consumption frequency for each food item on a daily, weekly, monthly, or yearly basis; portion sizes of consumed foods, reported in household measures, were then converted to daily grams of food intake. Considering that the Iranian Food Composition Table (FCT) is incomplete and has limited data on the nutrient content of raw foods and beverages, we used the United States Department of Agriculture (USDA) FCT. However, the Iranian FCT was used for local food items not listed in the USDA FCT.

#### Calculation of indices

We used participants’ dietary data derived from FFQ to determine dietary and lifestyle insulinemic scores. Because the intake of alcoholic drinks such as wine and liquor is unusual in the Iranian population due to religious considerations and the amount of their consumption has not been reported in the TLGS study; therefore, these food components were not considered to calculate the score of insulinemic indices. The calculation methods of four insulinemic indices, including EDIH, EDIR, ELIH, and ELIR, have been presented elsewhere [15]. As there are no food items as low-energy beverages and cream soup in our FFQ, we excluded these food items in the calculation of indices. The dietary and lifestyle insulinemic indices used in this study encompassed the following components:

##### EDIH

Processed meat (sausage), red meat (beef or lamb), poultry (chicken or turkey with or without skin), margarine, fish (canned tuna or fish), French fries, high-energy beverages (cola with sugar, fruit punch drinks, carbonated beverages with sugar), low-fat dairy products (skimmed or low-fat milk and yogurt), eggs, and tomatoes were considered as components of EDIH with a positive association. Also, green leafy vegetables (spinach, lettuce, or cabbage), whole fruits, coffee, and high-fat dairy products (whole milk, cream, cream cheese, and other cheese) were the OTHER components of EDIH with the inverse association.

##### EDIR

Margarine, refined grains, red meat, processed meat, tomatoes, fish, other vegetables, fruit juice with a positive association, green leafy vegetables, coffee, nuts, high-fat dairy products, and dark yellow vegetables with inverse association were considered as components of EDIR.

##### ELIH

BMI, margarine, butter, fruit juice (apple juice, cantaloupe juice, orange juice, or other fruit juice), and red meat with a positive association and also physical activity, coffee, high-fat dairy products, whole fruit, snacks, and salad dressing with the inverse association were considered as components of ELIH.

##### ELIR

BMI, red meat, processed meat, margarine, refined grains, other vegetables, tomatoes, potatoes, fruit juice, and tea with a positive association and also physical activity, green leafy vegetables, coffee, and high-fat dairy products with the inverse association were the components of ELIR.

To calculate the scores of the above-mentioned insulinemic indices, the food groups’ daily intakes (serving size) and lifestyle factors values were multiplied by specific proposed regression coefficients for weighting. Finally, all weighted food group intakes, BMI, and PA values were summed and then divided by 1000 to reduce the magnitude of scores, which eases the description and interpretation of results.

#### Statistical analyses

All statistical analyses of the current study were performed using the Statistical Package for Social Sciences (Version 20.0; SPSS, Chicago, IL). We used Kolmogorov–Smirnov test and histogram chart to assess the normality of variables. Baseline characteristics of the study population are reported as the mean ± SD or median (25–75 interquartile) for quantitative variables and percentages for qualitative variables. Individuals were classified according to EDIH and ELIH quintiles cut-off points. Chi-square and linear regression were used to test for trends of categorical and continuous variables across quintiles of EDIH and ELIH (as the median value in each quintile), respectively.

Individuals’ person-time (person-year) and duration of follow-up (in year) were calculated from baseline to the time at which an event (definitive diagnosis of CKD based on the EPI-eGFR level) occurred for the first time (event date), or the last date of follow up examination, whichever occurred first. The event date of occurrence of the CKD was determined as mid-time between the date of the follow-up visit at which the CKD was detected for the first time and the most recent follow-up visit preceding the diagnosis. For participants that remained healthy, their last CKD assessment from the baseline was considered for calculation of the follow-up period, and the exact time between the baseline date and last CKD assessment was computed as the follow-up period.

Multivariable Cox regression models were used with CKD as the dependent variable and EDIH, EDIR, ELIH, and ELIR as independent variables to estimate the risk of incident outcomes. Using Cox regression models, we reported the hazard ratios (HRs) and 95% confidence intervals (CIs). The first quintile of each above-mentioned insulinemic potential of diet and lifestyle indices was considered the reference group.

The association of EDIH and EDIR with HR of CKD was determined based on two logistic regression models that were included: a) model 1 (adjusted for age, sex) and b) model 2 (adjusted for model 1 and body mass index, smoking, physical activity, education level, baseline eGFR, energy intake, hypertension, and type 2 diabetes). For ELIH and ELIR, logistic regression analyses were fulfilled in two models: a) model 1 (adjusted for age and sex) and b) model 2 (adjusted for model 1 and energy intake, smoking, education level, baseline eGFR, hypertension, and type 2 diabetes). *P*-values < 0.05 were considered to be statistically significant.

We also computed restricted cubic splines with 5 knots based on equal sample sizes to test the shape of the association between each insulinemic index (EDIH, ELIH, EDIR, and ELIR) as a continuous variable and the risk of CKDs. The statistical significance of nonlinearity was assessed by comparing the spline model with the linear model, and *P* values of< 0.05 were considered as the statistically significant nonlinear association between the insulinemic indices with CKD. Statistical significance of linearity was tested by comparing the linear model to the model, including only the covariates, both using likelihood ratio tests.

## Results

Study participants’ (54.3% females) mean ± SD age and BMI were 37.8 ± 12.8 and 26.8 ± 4.7, respectively. The median (IQR) ELIH, ELIR, EDIH, and EDIR in participants were 1.31 (1.13–1.50), 4.14 (2.98–5.79), 0.17 (0.08–0.31), and 0.69 (0.47–0.99), respectively. During the 6.03 years of follow-up, 1216 incident cases (20.1%) of CKD was identified (the incidence rate = 260 per 10.000 person-years) among all study population.

The baseline characteristics and dietary intakes of participants according to the quintiles of ELIH score are presented in Table 1. Subjects in the highest ELIH score quintiles were more likely to be female, older, smoked less, and had lower physical activity and academic education levels than those in the lowest quintiles of ELIH (*P* < 0.05). Additionally, BMI, serum creatinine, and percentage of T2DM and HTN were increased significantly across ELIH score quintiles, whereas the level of eGFR was decreased (*P* < 0.001). Furthermore, participants in the highest quintile of ELIH score had higher intakes of energy, total fat, margarine, butter, and red meat, but lower intakes of fruits juice, whole fruits, coffee, snacks, salad dressing, and high-fat dairy products compared to those in the lowest quintile of ELIH.

**Table 1:** Baseline characteristics of participants according to quintiles (Q) of the empirical lifestyle index for hyperinsulinemia

	Empirical lifestyle index for hyperinsulinemia					*P* Value^*^
	Q1	Q2	Q3	Q4	Q5	
Age (years)	32.8 ± 13.3	37.1 ± 13.2	39.0 ± 12.1	40.1 ± 12.1	40.5 ± 11.6	< 0.001
Men (%)	45.9	48.3	48.6	48.2	38.0	< 0.001
Body mass index (kg/m2)	21.7 ± 2.7	24.7 ± 2.4	26.6 ± 2.5	28.7 ± 2.8	32.2 ± 4.7	< 0.001
Smoking (%)	13.2	12.4	12.5	13.2	10.7	0.335
Physical activity (MET/hour/week)	82.9 (38.1–125.0)	71.4 (27.0–107.9)	64.9 (23.3–103.9)	59.9 (20.8–102.2)	51.3 (15.9–90.0)	< 0.001
Academic education, (%)	25.1	27.3	26.0	23.6	18.5	< 0.001
Creatinine (mg/dl)	1.01 ± 0.14	1.02 ± 0.14	1.02 ± 0.15	1.03 ± 0.14	1.01 ± 0.14	0.154
Glomerular filtration rate (mL/min/1.73 m2)	83.9 ± 12.6	80.6 ± 12.0	79.2 ± 12.1	77.7 ± 11.7	77.5 ± 12.1	< 0.001
Hypertension (%)	4.1	6.9	7.8	13.3	15.0	< 0.001
Diabetes (%)	2.0	3.0	4.7	4.6	6.8	< 0.001
Empirical dietary index for hyperinsulinemia	0.17 ± 0.13	0.19 ± 0.17	0.20 ± 0.18	0.20 ± 0.24	0.35 ± 0.30	< 0.001
Empirical lifestyle index for hyperinsulinemia	0.95 ± 0.11	1.16 ± 0.04	1.31 ± 0.03	1.46 ± 0.05	1.75 ± 0.19	< 0.001
**Nutrient Intake**						
Energy(Kcal/d)	2387 ± 724	2329 ± 696	2306 ± 702	2310 ± 700	2431 ± 748	< 0.001
Carbohydrate(% of energy)	59.3 ± 6.7	59.0 ± 6.8	58.6 ± 11.8	58.2 ± 6.7	56.1 ± 6.9	< 0.001
Protein(% of energy)	14.0 ± 2.7	14.4 ± 2.9	15.0 ± 1.8	14.5 ± 2.7	14.6 ± 3.0	0.066
Fat(% of energy)	29.8 ± 6.0	29.9 ± 6.0	31.0 ± 6.0	30.3 ± 6.7	32.3 ± 7.0	< 0.001
**Food groups**						
Red meat (serving/week)	0.49 (0.28–0.77)	0.56 (0.28–0.91)	0.63 (0.35–1.19)	0.70 (0.42–1.26)	0.91 (0.49–1.89)	< 0.001
Fruit juice(serving/week)	0.12 (0.07–0.91)	0.28 (0.07–0.70)	0.21 (0.02–0.54)	0.28 (0.07–0.70)	0.21 (0.03–0.70)	0.352
Coffee(serving/d)	0.00 (0.00–0.03)	0.00 (0.00–0.03)	0.00 (0.00–0.02)	0.00 (0.00–0.02)	0.00 (0.00–0.02)	0.030
Butter and Margarine (serving/d)	0.16 (0.00–0.71)	0.16 (0.01–0.71)	0.25 (0.01–1.07)	0.41 (0.02–1.42)	0.71 (0.04–2.50)	< 0.001
Whole fruit(serving/d)	1.65 (0.83–2.72)	1.54 (0.80–2.65)	1.50 (0.78–2.40)	1.39 (0.74–2.30)	1.41 (0.75–2.32)	< 0.001
High-fat dairy products(serving/d)	1.31 (0.85–2.12)	1.23 (0.82–1.96)	1.17 (0.72–1.79)	1.11 (0.69–1.65)	1.09 (0.66–1.59)	< 0.001
Snacks(serving/d)	0.17 (0.03–0.57)	0.14 (0.02–0.41)	0.14 (0.02–0.36)	0.13 (0.01–0.31)	0.14 (0.02–0.35)	< 0.001
Salad dressing(serving/d)	0.16 (0.06–0.42)	0.15 (0.06–0.34)	0.14 (0.04–0.31)	0.14 (0.04–0.29)	0.14 (0.05–0.31)	< 0.001

Data represented as mean ± standard deviation, or median (interquartile) for continuous variables and number and percent for categorical variables

^*^Chi-square and linear regression were used to test the trend of continuous and categorical variables across quintiles of the empirical dietary index for hyperinsulinemia (as the median value in each quartile), respectively

We also showed individuals’ baseline characteristics and dietary intakes according to the quintiles of the ELIR score in Table 2. The mean BMI, eGFR, physical activity, % of male subjects, and % of smoking were increased across quintiles of the ELIR score, whereas the mean age was reduced across quintiles of this score. Total energy, dietary intakes of carbohydrates, protein, refined grains, red meats, margarine, tomatoes, and potatoes significantly increased across quintiles of ELIR score (*P* < 0.001). However, the intakes of total fats, tea, high-fat dairy products, and green leafy vegetables were decreased across quintiles of this score (*P* < 0.001).

**Table 2:** Baseline characteristics of participants according to quintiles (Q) of the empirical lifestyle index for insulin resistance

	Empirical lifestyle index for insulin resistance					*P* Value*
	Q1	Q2	Q3	Q4	Q5	
Age (years)	40.0 ± 13.4	38.4 ± 12.8	36.9 ± 12.5	37.7 ± 12.3	36.5 ± 12.6	< 0.001
Men (%)	37.8	40.2	42.8	48.3	60.0	< 0.001
Body mass index (kg/m2)	25.9 ± 4.1	27.0 ± 4.7	26.7 ± 4.8	27.2 ± 4.8	27.8 ± 5.0	< 0.001
Smoking (%)	10.3	10.5	11.6	13.2	16.2	< 0.001
Physical activity (MET/hour/week)	63.5 (20.8–105.7)	62.5 (22.2–102.7)	63.1 (23.6–105.9)	68.6 (26.9–107.8)	71.4 (27.7–108.3)	0.001
Academic education, (%)	23.1	22.7	27.2	23.1	24.5	0.078
Creatinine (mg/dl)	1.00 ± 0.14	1.01 ± 0.14	1.02 ± 0.14	1.03 ± 0.15	1.06 ± 0.14	< 0.001
Glomerular filtration rate (mL/min/1.73 m2)	78.4 ± 12.6	79.2 ± 12.5	80.4 ± 12.3	79.7 ± 12.1	81.1 ± 12.2	< 0.001
Hypertension (%)	8.2	10.2	8.6	9.4	10.7	0.227
Diabetes (%)	4.6	4.3	4.0	4.1	4.1	0.933
Empirical dietary index for insulin resistance	0.37 ± 0.23	0.58 ± 0.27	0.71 ± 0.28	0.89 ± 0.30	1.38 ± 0.47	< 0.001
Empirical lifestyle index for insulin resistance	2.31 ± 0.34	3.20 ± 0.24	4.17 ± 0.32	5.43 ± 0.42	8.88 ± 2.62	< 0.001
**Nutrient Intake**						< 0.001
Energy(Kcal/d)	1984 ± 634	2239 ± 666	2307 ± 687	2431 ± 664	2801 ± 670	< 0.001
Carbohydrate(% of energy)	57.3 ± 7.4	57.9 ± 6.8	57.4 ± 6.5	58.7 ± 11.9	59.8 ± 6.4	< 0.001
Protein(% of energy)	15.5 ± 3.7	14.9 ± 3.6	14.4 ± 2.8	14.3 ± 11.4	13.2 ± 2.1	< 0.001
Fat(% of energy)	31.4 ± 6.9	30.7 ± 6.4	31.3 ± 6.3	31.0 ± 6.0	28.9 ± 6.4	< 0.001
**Food groups**						
Refined grains(serving/d)	1.31 (0.96–1.61)	2.30 (1.92–2.68)	3.59 (3.20–4.03)	5.17 (4.73–5.72)	8.62 (7.32–11.01)	< 0.001
Red meat (serving/week)	0.21 (0.28–0.84)	0.56 (0.35–0.98)	0.70 (0.35–1.26)	0.77 (0.42–1.26)	0.84 (0.56–1.47)	< 0.001
Tomatoes(serving/d)	0.47 (0.31–1.11)	0.63 (0.31–1.11)	0.63 (0.31–1.11)	0.63 (0.31–1.11)	0.63 (0.31–1.11)	< 0.001
Fruit juice(serving/d)	0.03 (0.00–0.09)	0.04 (0.01–0.10)	0.04 (0.01–0.10)	0.04 (0.01–0.10)	0.04 (0.01–0.11)	0.644
Potatoes(serving/d)	0.06 (0.01–0.09)	0.06 (0.02–0.12)	0.06 (0.02–0.12)	0.06 (0.03–0.12)	0.06 (0.04–0.15)	< 0.001
Processed meat(serving/week)	0.06 (0.01–0.14)	0.10 (0.02–0.24)	0.13 (0.04–0.30)	0.13 (0.05–0.32)	0.16 (0.08–0.42)	< 0.001
Other vegetables(serving/d)	1.70 (1.01–3.12)	2.00 (1.28–2.97)	1.91 (1.15–2.87)	1.91 (1.10–3.12)	1.84 (1.08–2.74)	0.079
Tea(serving/d)	2.08 (1.04–3.12)	2.08 (1.04–3.12)	2.08 (1.04–3.12)	2.08 (1.04–3.12)	2.08 (1.04–3.12)	< 0.001
Coffee(serving/week)	0.01 (0.00–0.13)	0.02 (0.00–0.13)	0.03 (0.00–0.24)	0.02 (0.00–0.24)	0.03 (0.00–0.25)	0.600
High-fat dairy products(serving/d)	1.27 (0.85–1.97)	1.19 (0.78–1.88)	1.18 (0.72–1.91)	1.14 (0.72–1.85)	1.10 (0.66–1.61)	< 0.001
Green leafy vegetables(serving/d)	0.33 (0.15–0.63)	0.34 (0.17–0.64)	0.29 (0.14–0.56)	0.27 (0.13–0.52)	0.24 (0.11–0.48)	< 0.001

Data represented as mean ± standard deviation, or median (interquartile) for continuous variables and number and percent for categorical variables

*Chi-square and linear regression were used to test the trend of continuous and categorical variables across quintiles of the empirical dietary index for insulin resistance (as the median value in each quartile), respectively

Table 3 shows the results on the HR of CKD according to quintiles of EDIH, EDIR, ELIH, and ELIR. Based on the age and sex-adjusted model, compared to participants in the first quintile of EDIR, ELIH, and ELIR, participants in the fifth quintile of these indices had a higher risk of incident CKD by 28, 34, and 24%, respectively [EDIR (HR = 1.28; 95%CI: 1.07–1.52), ELIH (HR = 1.34; 95%CI: 1.09–1.64), and ELIR (HR = 1.24; 95%CI: 1.04–1.48)]. Also, we observed a significant increase in HR of CKD per unit increase in the quintile of EDIR (P for trend: 0.006), ELIH (P for trend: 0.005), and ELIR (P for trend: 0.015) based on the age and sex-adjusted model. However, there was no significant association between the higher score of EDIH and the risk of developing CKD (HR = 1.06; 95%CI: 0.87–1.25).

**Table 3:** The association between the lifestyle and dietary insulinemic indices and incidence of chronic kidney disease: the Tehran Lipid and Glucose Study

	Lifestyle and dietary insulinemic indices				
	Q1	Q2	Q3	Q4	Q5
**EDIH**					
Median score	0.009	0.096	0.170	0.272	0.516
Follow up period	7.66 ± 2.84	7.87 ± 2.68	7.67 ± 2.75	7.82 ± 2.66	7.77 ± 2.67
person-years	9263.2	9500.2	9274.0	9456.8	9395.4
Case/Total	304/1209	247/1207	243/1209	221/1208	200/1208
Incidence rate (10.000 person year)	328	259	262	233	212
Model 1^a^	1.00 (Ref)	0.87 (0.73–1.03)	1.02 (0.86–1.20)	1.09 (0.91–1.29)	1.06 (0.87–1.25)
Model 2^b^	1.00 (Ref)	0.87 (0.73–1.05)	1.03 (0.86–1.23)	1.11 (0.92–1.33)	1.08 (0.89–1.31)
**EDIR**					
Median score	0.312	0.517	0.695	0.918	1.386
Follow up period	7.81 ± 2.86	7.76 ± 2.71	7.70 ± 2.73	7.85 ± 2.62	7.66 ± 2.69
person-years	9441.5	9393.1	9311.5	9490.4	9256.6
Case/Total	242/1208	218/1208	238/1209	251/1209	266/1208
Incidence rate (10.000 person year)	261	229	255	267	281
Model 1^a^	1.00 (Ref)	1.03 (0.86–1.22)	1.08 (0.91–1.29)	1.05 (0.87–1.25)	1.28 (1.07–1.52)
Model 2^b^	1.00 (Ref)	1.01 (0.84–1.21)	1.14 (0.95–1.38)	1.02 (0.84–1.29)	1.29 (1.06–1.57)
**ELIH**					
Median score	0.98	1.17	1.31	1.45	1.69
Follow up period	7.79 ± 3.04	7.87 ± 2.64	7.77 ± 2.81	7.63 ± 2.76	7.47 ± 2.79
person-years	9552.1	9335.2	9237.9	9051.7	8877.0
Case/Total	145/1187	225/1186	248/1188	278/1186	303/1187
Incidence rate (10.000 person year)	151	241	268	307	341
Model 1^a^	1.00 (Ref)	1.13 (0.92–1.39)	1.23 (1.00–1.51)	1.18 (0.97–1.44)	1.34 (1.09–1.64)
Model 2^c^	1.00 (Ref)	1.14 (0.91–1.41)	1.25 (1.01–1.55)	1.20 (0.97–1.48)	1.35 (1.10–1.67)
**ELIR**					
Median score	2.37	3.20	4.14	5.38	8.06
Follow up period	7.83 ± 3.01	7.85 ± 2.81	7.76 ± 2.85	7.73 ± 2.68	7.67 ± 2.65
person-years	9323.1	9212.6	9187.3	9100.0	9226.0
Case/Total	219/1187	242/1186	226/1188	234/1186	277/1187
Incidence rate (10.000 person year)	237	262	245	257	297
Model 1^a^	1.00 (Ref)	1.06 (0.89–1.26)	1.18 (0.98–1.40)	1.13 (0.95–1.35)	1.24 (1.04–1.48)
Model 2^c^	1.00 (Ref)	1.07 (0.90–1.29)	1.17 (0.97–1.41)	1.20 (1.00–1.45)	1.24 (1.02–1.51)

*Abbreviations*: *EDIH* Empirical dietary index for hyperinsulinemia, *ELIH* Empirical lifestyle index for hyperinsulinemia, *EDIR* Empirical dietary index for insulin resistance, *ELIR* Empirical lifestyle index for insulin resistance

^a^ Model 1: adjusted for age and sex

^b^ Model 2: additionally adjusted for model 2 and body mass index, smoking, physical activity, education level, baseline eGFR, energy intake, hypertension, and type 2 diabetes

^c^ Model 2: additionally adjusted for model 2 and energy intake, smoking, education level, baseline eGFR, hypertension, and type 2 diabetes

In the multivariable-adjusted model, after adjusting for potential confounding factors, individuals in the highest quintile of EDIR (HR = 1.29; 95% CI: 1.06–1.57), ELIH (HR = 1.35; 95%CI: 1.10–1.67), and ELIR (HR = 1.24;95%CI:1.02–1.51) had significantly higher risk of incident CKD than those in the lowest quintile of these indices. Also, based on the final cox regression model, our findings showed that there is a significant increase in HR of CKD per unit increase in the quintile of EDIR (P for trend: 0.016), ELIH (P for trend: 0.006), and ELIR (P for trend: 0.026). However, no significant relationship was observed between EDIH and CKD risk, based on a fully adjusted model (HR = 1.08; 95%CI: 0.89–1.31).

Comparing the spline with linear modes showed no significant non-linear association between insulin indices and CKD incidence. Also, except for ELIR, other indices showed any significant linear relationship with CKD [EDIH (P-nonlinearity = 0.739 and P-linearity = 0.567), EDIR (P-nonlinearity = 0.147 and P-linearity = 0.174), ELIH (P-nonlinearity = 0.649 and P-linearity = 0.228), and ELIR (P-nonlinearity = 0.281 and P-linearity = 0.004)]. Models were adjusted for age, sex, BMI (only for EDIH and EDIR), physical activity (only for EDIH and EDIR), smoking, education level, baseline eGFR, energy intake, hypertension, and type 2 diabetes.

## Discussion

In this population-based cohort study, we determined the insulinemic potential of diet and lifestyle indices, including EDIH, EDIR, ELIH, and ELIR, and assessed their relationship with the risk of developing CKD, independent of potential confounders, among the adult population. We showed that higher EDIR, ELIH, and ELIR scores were associated with a higher risk of incident CKD by 29, 35, and 24%, respectively, whereas no significant association was found between EDIH and risk of CKD.

A growing body of evidence suggests that insulin metabolism-related disorders such as central obesity, IR, and hyperinsulinemia can contribute to the progression and development of kidney dysfunction and an increased risk of CKD [31]. On the other hand, some reports revealed that higher dietary and lifestyle insulinemic potential is associated with an increased risk of adiposity, IR, hyperinsulinemia, and type 2 diabetes [19, 22]. Considering that the above-mentioned metabolic outcomes are each predisposing factors for an increased risk of kidney impairment, we hypothesized that a high insulinemic diet and lifestyle could also play a significant role in the pathogenesis of CKD. Although there is no study on the association of the insulinemic potential of diet and lifestyle with the risk of CKD, our findings are in agreement with the results of most previous studies supporting a direct link between a higher insulinemic diet and lifestyle with the risk of chronic metabolic diseases. A cohort study in the framework of TLGS indicated that higher scores of EDIR, ELIR, and ELIH were associated with an increased risk of T2DM, while no significant association was observed between EDIH and the risk of T2DM [21]. Mokhtari et al. suggested that adherence to a lifestyle with a higher score of ELIH may be associated with an increment in the risk of IR and hyperinsulinemia. However, no significant association was found between a high insulinemic diet and the risk of insulin-related disorders [19]. Additionally, a cohort study on a large sample of US female nurses showed that higher EDIH and ELIH were related to a higher risk of colorectal cancer in the young population [16]. Furthermore, the Nurses’ Health Study findings reported that adherence to a dietary pattern with higher insulinemic potential was associated with a higher risk of T2DM [22]. In general, the results of previous studies indicate that dietary and lifestyle patterns contributing to IR and hyperinsulinemia can play a remarkable role in the pathogenesis of metabolic disorders and their related chronic diseases, therefore, our findings on the positive association of high insulinemic diet and lifestyle with CKD risk can be logical and valuable findings.

Based on our main results, there is no significant association between a higher score of EDIH and the risk of CKD. Non-significant results regarding EDIH score with risk of metabolic disorders such as IR and T2D incident have also been seen in previous studies conducted on the Iranian population. Contrary to the results of studies conducted on other people, among the Iranian people, the EDIH score has indicated low potency in predicting the risk of metabolic disorders such as IR [19], T2DM [21], and CKD. This inconsistency in results can be mainly justified by the low consumption of dietary components of EDIH in our study population, which subsequently leads to lower estimated scores for the EDIH index among individuals. Also, in the current study, individuals’ intakes for the food components of EDIH were close to each other and did not have high dispersion, therefore, the estimated EDIH score in our study population had a narrow range. Moreover, the EDIH index was initially developed and validated in different populations. Therefore variations in diet patterns and genetic background exist in comparison to our population, which could potentially be responsible for this consistency in our results with others.

Our results suggest that hyperinsulinemia may be a potential mechanism linking dietary and lifestyle insulinemic indices to CKD development. The insulinemic effect of inappropriate food choices such as higher consumption of red and processed meat, margarine, refined grains, and sweetened beverages and lower consumption of vegetables, legumes, whole grain, and dairy products in combination with high body fat and sedentary life, as main parts of lifestyle, may play a key role in increasing chronic insulin secretion. It has been suggested that high chronic insulin secretion leads to beta cell dysfunction, increased central obesity, and a higher risk of IR [19, 32]. Consequently, hyperinsulinemia, increased adiposity, and IR could result in developing kidney dysfunction and increased risk of CKD during a long period through the increments in glomerular hyperfiltration, endothelial dysfunction, albumin excretion, and inducing vascular permeability [7, 8, 33]. Lastly, IR may cause glomerulosclerosis or atherosclerosis-related kidney impairment in the elderly via inducing oxidative stress and endothelial dysfunction [34, 35].

Our study has several main strengths. To the best of our knowledge, this is the first study to investigate the association of the insulinemic potential of diet and lifestyle indices, including EDIH, EDIR, ELIH, and ELIR, with the risk of CKD in the adult population. The prospective design, long-term follow-up time, and as well as relatively large sample size are the other major strengths of this study. Also, in the current study, we used valid and reliable questionnaires to assess the data on participants’ dietary intakes and physical activity levels. Despite these strengths, this study is not without limitations. First, some measurement errors are inevitable because of using FFQ for dietary assessment; however, similar to other epidemiological studies, we have used a valid and reliable questionnaire, which minimizes this error. Second, Similar to most epidemiologic studies, the serum creatinine was measured only once in our study to detection of CKD; however, it has been recommended that creatinine be measured 3 times to enhance the accuracy of detecting CKD. We did not have data for the measurement of microalbuminuria, which could help us determine early kidney damage in participants based on insulin index scores, however, we used serum creatinine and eGFR to determine the occurrence of CKD in participants, which is a common method to determine CKD in epidemiological studies. Also, the level of plasma insulin and its related indicators did not measure in the participants, which could help determine the insulinemic effect of diet and lifestyle. Furthermore, although we controlled the effects of major confounding variables in our final statistical analysis, there may still be residual or unmeasured confounders, such as fluid intake and family history of CKD effects which cannot be ruled out.

## Conclusions

Results of the current study indicated that dietary patterns with a high EDIR score and a lifestyle with higher ELIH and ELIR scores may be associated with an increased risk of CKD, while no significant association was reported between EDIH and the risk of developing CKD. Further prospective studies with long-term follow-up are recommended to investigate the possible role of the insulinemic potential of diet and lifestyle in the risk of T2DM and CKD among other populations.

## Data Availability

The datasets analyzed in the current study are available from the corresponding author on reasonable request.

## Abbreviations

BMI: Body mass index
CKD: Chronic kidney disease
DBP: Diastolic blood pressure
EDIH: Empirical dietary indexes for hyperinsulinemia
EDIR: Empirical dietary indexes for IR
ELIH: Empirical lifestyle indices for hyperinsulinemia
ELIR: Empirical lifestyle indices for IR
EPI: Epidemiology Collaboration equation
FCT: Iranian Food Composition Table
FFQ: Food frequency questionnaire
FPG: Fasting blood glucose
GFR: Glomerular filtration rate
HTN: Hypertention
IR: Insulin resistance
MAQ: Modifiable activity questionnaire
MET: Metabolic equivalent
SBP: Systolic blood pressure
T2DM: Type 2 diabetes mellitus
TLGS: Tehran Lipid and Glucose Study
USDA: United States Department of Agriculture
WC: Waist circumference
